# Combined analysis and miRNA expression profiles of the flowering related genes in common wild rice (*oryza rufipogon* Griff.)

**DOI:** 10.1007/s13258-018-0688-y

**Published:** 2018-04-21

**Authors:** Jiao Wang, Yan Long, Jingwen Zhang, Mande Xue, Gege Huang, Ke Huang, Qianhua Yuan, Xinwu Pei

**Affiliations:** 1 Institute of Biotechnology, Chinese Academy of Agricultural Sciences, Beijing, China; 2 Hainan Key Laboratory for Sustainable Utilization of Tropical Bioresources, College of Agriculture, Hainan University, Haikou, China

**Keywords:** *Oryza rufipogon* Griff., Transcriptome, MiRNAs, Flowering, Combined analysis, High-throughput sequencing

## Abstract

Common wild rice (*Oryza rufipogon* Griff.) is the most closely related ancestral species to Asian cultivated rice (*Oryza sativa* L.). It contains various valuable traits with regard to tolerance to cold, drought and salinity, flowering diversity and many quantitative trait loci with agronomic important traits. Flowering is one of the most important agronomic traits. However, flowering-related transcriptome and how to be regulated by miRNAs have not been estimated in *O.rufipogon*. To identify how the genes and miRNAs regulating flowering in *O.rufipogon*. Three *O.rufipogon* RNA libraries, two vegetative stages (CWRT-V1 and CWRT-V2) and one flowering stage (CWRT-F2) were constructed using leaves tissue and sequenced using Illumina deep sequencing. 27,405, 27,333, 28,979 unique genes were obtained after mapping to the reference genome from CWRT-V1, CWRT-V2 and CWRT-F2, respectively. Then differentially expressed genes (DEGs) were screened and got 1419 unique genes are likely to involve in flower development. Detailed information showed that MADS box and floral meristem identity genes, such as *MADS 1, MADS14, Hd1* are involved in common wild rice. Then, combined analysis of miRNA and mRNA expression profiles was performed. Twenty three known miRNA-mRNA pairs and five new candidates were presented an anti-correlationship. Interestingly, 12 miRNAs were negatively correlated with 20 mRNAs encoding flowering-related proteins, indicating that miRNAs regulated target genes to promote flowering in CWRT-F2 group. The results provided here genomic resources for flowering related genes and how these flowering genes were regulated by miRNAs in common wild rice.

**Electronic supplementary material:**

The online version of this article (10.1007/s13258-018-0688-y) contains supplementary material, which is available to authorized users.

## Introduction

Flowering is an important transition stage between the vegetative to reproductive stage. Appropriate flowering time is necessary for crop reproduction (Jarillo and Pineiro 2011). The biological phenomenon and genetic mechanism that govern flowering in plants have been investigated for more than 100 years (Kovach et al. 2007). The floral transition is closely related to grain production and relies mainly on different day-length in rice. There are two pathways to regulate photoperiodic flowering: the *Hd1-Hd3a* and *Ghd7-Ehd1-Hd3a*/*RFT1* module (Izawa 2007). In addition to the core regulators, several other genes have been identified by using classical genetic methods as flowering time regulatory factors, for example, *OsGI* (Hayama et al. 2003), *OsMADS14,15* (Kyozuka et al. 2000), *OsMADS50* (Lee et al. 2004; Yoshida and Nagato 2011), *Days to heading 7* (*DTH7*) (Gao et al. 2014), *Heading Date Repressor1* (*HDR1*) (Sun et al. 2016), *Heading date 18* (*Hd18*) (Shibaya et al. 2016).

*O. rufipogon* Griff., regarded as the closest ancestral relative to Asian cultivated rice (*O. sativa* L.), is distributed only at lower latitudes in the tropical and subtropical regions of Asia and Oceania (Khush 1997; Vaughan et al. 2008). Floral transition under short day conditions at lower latitudes plays a more important role than at higher latitudes. Similar to cultivated rice, the short-day conditions that govern *Hd1* response play a role for common wild rice in tropical regions (Izawa 2007). Several studies have focused on flowering time traits as well as the genetic basis for photoperiod-controlled flowering response. For example, Dong et al. (2010) investigated the flowering time of 11 populations of *O.rufipogon* from Hainan Island in China and found that they normally flowered in September–December, with flowering time lasting 2–3 months (Dong et al. 2010). Hagiwara et al. (2009) studied polymorphisms of the *FT-like* gene between common wild rice and cultivated rice, and found more allelic variations existing in *Hd3a* and *RFT1* from common wild rice than in those genes in cultivated rice (Hagiwara et al. 2009). As the most useful genetic resource for Asian cultivated rice, *O.rufipogon* has been investigated for valuable traits. Primarily, research has focused on cloning of useful genes, such as the grain shattering gene *SH4* (Li et al. 2006; Lin 2007), *qSH1* (Konishi et al. 2006). Another main focus of research in *O. rufipogon* was genomic analysis, such as analyzing genetic diversity (Rakshit et al. 2007), genome domestication investigation (Kovach et al. 2007). To identify useful genetic resources, single gene isolation and analysis has been the standard mode for operation, while little work was done by using large-scale differential gene expression.

For gene expression analysis, real-time PCR technology is regarded as the standard method. However, improvements to high-throughput sequencing technology are driving innovations in transcriptomic sequencing and analysis, thus pushing this technology to become a commonly used method. Using high-throughput sequencing, researchers have altered our view of the extent and complexity of eukaryotic transcriptomes, facilitated the discovery of novel genes (Qi et al. 2012; Wang et al. 2009). For example, several differentially-transcribed genes related to floral transition were identified in Chrysanthemum by Illumina sequencing (Ren et al. 2016). Harrop et al. (2016) used gene expression profiling to analyze genes that participated in reproductive meristem development in early rice inflorescences (Harrop et al. 2016). Along with RNA-seq, digital gene expression (DGE) is becoming a common method for measuring and comparing the expression of thousands of genes at once. From these data, useful candidate genes can be selected for cloning based on a large set of genetic information. For example, Tang et al. (2011) used RNA-seq and DGE technology to identify candidate genes encoding enzymes responsible for the biosynthesis of novel secondary metabolites in a native plant, Siraitiagrosvenorii (Luohanguo), in China. Similarly, seven CYP450s and five UDPGs were selected as potential candidates for use in mogroside biosynthesis (Kovach et al. 2007; Rakshit et al. 2007; Tang et al. 2011).

Regulation of flowering is also carried out by microRNAs (miRNAs), a class of 20–24 nt, non-coding, small RNAs. These miRNAs down-regulate flowering-related genes via complementarity of the 9–11th nucleotide positions at the 5′ end of the miRNAs with the target gene (Jones-Rhoades et al. 2006; Spanudakis and Jackson 2014). MiR156 and miR172 are typical examples of miRNAs that coordinate regulation of vegetative and reproductive development in *Arabidopsis thaliana* and rice (Wang et al. 2015). In rice, miR156 targets the transcription factor *SQUAMOSA PROMOTER BINDING PROTEINLIKE* (*SPL*) gene family, and over-expression of miR156 results in many more tillers, delay of flowering, and small panicles (Wang et al. 2015). Therefore, miR156 negatively regulates inflorescence meristem differentiation and the initiation of reproductive branching. Conversely, miR172 targets *APETALA2* (*AP2*)-like transcription factors, and over-expression of miR172 promotes early flowering and severe reduction in panicle branching (Lee et al. 2014). Taken together, miR156 regulates vegetative development, while miR172 plays an important role in regulation of the reproductive stage. Both play a part in control of the transition from juvenile to flowering stage. It has been reported that overexpression of miR319, which targets TCP TFs in *Arabidopsis*, moderately delays flowering (Schommer et al. 2008). The miR171 family, well-conserved between species, targets *LOST MERISTEMS* (*LOM*) TFs. Overexpression of miR171 delays flowering in barley and rice (Curaba et al. 2013; Fan et al. 2015).

In previous studies, we identified common wild rice plants which collected from the same population but with different flowering time. Sub-populations were categorized based on flowering time phenotypes including a single-flowering sub-group, and a double-flowering sub-group (Chen et al. 2013).Then the allelic variations of flowering time related genes, such as *GI,Hd1*,and *Edh* were checked in the populations. The result showed that there was no genotypic differences between the two sub-populations in this set of genes, so we anticipated that there are other pathways or genes that control flowering time in the one-time flowering subgroup (Dong et al. 2012). In this study, we used transcriptome sequencing and digital gene expression (DGE) technology to investigate changes in gene expression between the single-flowering and double-flowering sub-groups in the vegetative and flowering stage. Analysis of the gene expression data revealed 1419 differentially expressed genes (DEGs) that are likely related to flowering. Then mRNA-miRNA combined analysis was applied to investigate the role of miRNA regulation of target genes in flowering in *O. rufipogon*.

## Materials and methods

### Plant materials

The common wild rice was collected from Gaozhou Common Wild Rice Protection Area in Guangdong province, and approved by Guangdong Province Agriculture Department. In the previous survey, we found that there were two groups of plants within the same population of the original habitat that differed in their flowering time. The single-flowering group flowered from October to December, and the double-flowering group flowered initially in April–June, and again from October to December (Chen et al. 2013). Individual specimen of these two sub-groups were planted in growth chamber at day/night temperatures of 32/28 °C (8 h day/16 h night) and a relative humidity of 65%. We then collected the leaf samples from the vegetative stage with six leaves of the single-flowering sub-group and the double-flowering sub-group, respectively. Meanwhile, the leaf samples from the flowering stage of the double-flowering sub-group were collected. All the samples were stored in liquid nitrogen for RNA extraction.

### RNA extraction and cDNA library preparation for transcriptome analysis

Total RNA was extracted with TRIzol Reagent (Invitrogen, 15596-026) according to the manufacturer’s instructions. RNA samples that met the requirements were used to construct the sequence libraries. mRNA sequencing samples were prepared using the RNA-seq sample preparation kit (Illumina, San Diego, CA). Following the Illumina manufacturer’s procedures, mRNA was purified from 10 mg of the pooled total RNA using polyT oligo-attached magnetic beads. Fragmentation buffer was added to disrupt the mRNA into short fragments. Reverse transcriptase and random primers were used to synthesize the first strand cDNA from the cleaved mRNA fragments. The second strand cDNA was synthesized using buffer, dNTPs, RNase H, and DNA polymerase I. The double strand cDNA was purified with QiaQuick PCR extraction kit (QIAGEN, Hilden, Germany) and washed with EB buffer for end repair and single nucleotide A(adenine) addition. Finally, sequencing adaptors were ligated to the fragments. The required fragments were purified by agrose gel electrophoresis and enriched by PCR to construct a cDNA library. The cDNA libraries were named with CWRT-V1 (leaf sample from one-flowering sub-group), CWRT-V2 (leaf sample from one-flowering sub-group) and CWRT-F2 (leaf sample from double-flowering sub-group).

### Transcriptome sequencing and bioinformatics analysis

The cDNA library was sequenced using the Illumina HiSeq2000 System. The raw data was filtered to remove adapter sequences, reads with > 10% unknown bases, and low-quality sequences (more than 50% of the read has a quality score ≤ 5). Clean reads were mapped to *Japonica* reference sequences (http://rice.plantbiology.msu.edu) using SOAP aligner/soap2 (Li et al. 2009). Mismatches of greater than two bases were excluded from the alignment. Clean data, mapped to only one gene in the reference database, were designated as unambiguous tags for annotation and expression level analysis. Gene expression was calculated by counting the reads mapped to the reference sequence, and for each gene the expression level was determined with the RPKM method (Mortazavi et al. 2008). The RPKM method is able to eliminate the influence of different gene length and sequencing discrepancy on the calculation of gene expression. Therefore, the calculated gene expression can be directly used for comparison of gene expression between samples. If there is more than one transcript for a gene, the longest one is used to calculate its expression level and coverage.

### Screening and analysis of differentially expressed genes

After calculating the gene expression level, the differentially expressed genes (DEGs) were screened by comparison of their expression levels. the method described by Audic and Claverie (1997) was used for DEG screening between two separate DGE libraries (Audic and Claverie 1997). The false discovery rate (FDR) method determined the P value threshold for multiple testing by controlling the FDR value. The criteria of FDR ≤ 0.001 and the absolute value of log2ratio ≥  1 were used to judge the significance of differences in gene expression. For GO enrichment analysis for functional significance, the hypergeometric test was applied to map all the differentially expressed genes to terms in the GO database, thus identifying GO terms significantly enriched for DEGs compared to the genome background. A corrected P value ≤ 0.05 was set as a threshold to identify the significant enrichment of GO terms in differential gene expression. The differentially expressed genes were also utilized in KEGG ontology (KO) enrichment analyses to further understand their biological functions. Pathway enrichment analysis identified significantly enriched metabolic or signal transduction pathways in DGEs, as compared with expression of the genome background. Pathways with Q value ≤ 0.05 were viewed as significantly enriched in DEGs.

### Validation of miRNAs and gene expression by quantitative real-time PCR

In general, miRNAs can pair with and negatively regulate several target genes. In our previous study, 44 flowering related miRNAs were identified by using the same populations (Chen et al. 2013). In this study, the combined analysis of mRNA and miRNA was applied. Quantitative real-time reverse transcription PCR (qRT-PCR) was performed to validate the mRNA and miRNA sequencing data. One microgram of total RNA was reverse transcribed into single-stranded cDNA using the Primescript RT reagent kit (TaKaRa, Dalian, China). The differently expressed miRNAs were verified by quantitative stem-loop-PCR as described in Chen et al. (2013). For qRT-PCR, the SYBR premix Ex Taq kit (TaKaRa, Dalian, China) was used for reactions run on an ABI 7500 Real-Time System (Applied Biosystems), with the first strand cDNA serving as the template. The *OsActin* (GenBank:AB047313) (Fang et al. 2008) gene was utilized as an internal control, and *U6* as the miRNA internal reference. The cDNA was serially diluted (50, 25, 12.5, 6.25 and 3.125 ng) and each cDNA was amplified by real-time PCR with the gene-specific primers using the SYBR green method. Each dilution was replicated three times. The mean of three replications was used in determining the absolute value of the slope of log (input amount) versus ΔCT. The relative quantitative method (ΔΔCT) was used to calculate the fold change of target genes (Quail et al. 2008). All reactions were performed using one biological sample with three technical replicates. The primers employed in the qRT-PCR are listed in Table S1.

## Results

### Statistics of RNA-seq sequence abundance in common wild rice

For three cDNA sequence libraries CWRT-V1, CWRT-V2, and CWRT-F2, the raw data were 7,046,714 reads, 7,231,932 reads, and 7,155,368 reads, respectively. The quality test showed that all the three libraries had 6,990,340(99.2%), 7,088,016 (98.01%) and 7,108,858 (99.35%) clean reads (Table 1).The sequencing saturation analysis showed that about 40% genes of the reference genome could be mapped by clean reads. After the clean reads were mapped to the reference genome by using SOAP aligner, it was found that 5,829,222 reads, 5,973,616 reads, and 5,790,541 reads for each library, respectively, could be mapped to the reference genome. Among them, 91% (5,329,222), 92.3% (5,510,755) and 91.8% (5,319,917) reads for each library, respectively, were uniquely matched reads. After the total clean reads were mapped to genes from the reference genome, there were 27,405 unique genes for CWRT-V1, 27,333 for CWRT-V2, and 28,979 for CWRT-F2, respectively.

**Table 1:** Summary information of DGE sequencing

Category	CWRT-V1		CWRT-V2		CWRT-F2	
	Reads number	%	Reads number	%	Reads number	%
Total reads	7,046,714	100.00	7,231,932	100.00	7,155,368	100.00
Total basepairs	345,288,986	100.00	354,364,668	100.00	350,613,032	100.00
Total mapped reads	5,859,055	83.15	5,973,616	82.60	5,790,541	80.93
Perfect match	4,636,536	65.80	4,706,869	65.08	4,539,940	63.45
≤ 3 bp mismatch	1,222,519	17.35	1,266,747	17.52	1,250,601	17.48
Unique match	5,329,222	75.63	5,510,755	76.20	5,319,917	74.35
Multi-position match	529,833	7.52	462,861	6.4	470,624	6.58
Total unmapped reads	1,187,659	16.85	1,258,316	17.40	1,364,827	19.07

### Identification of differentially expressed genes

**Fig. 1:**
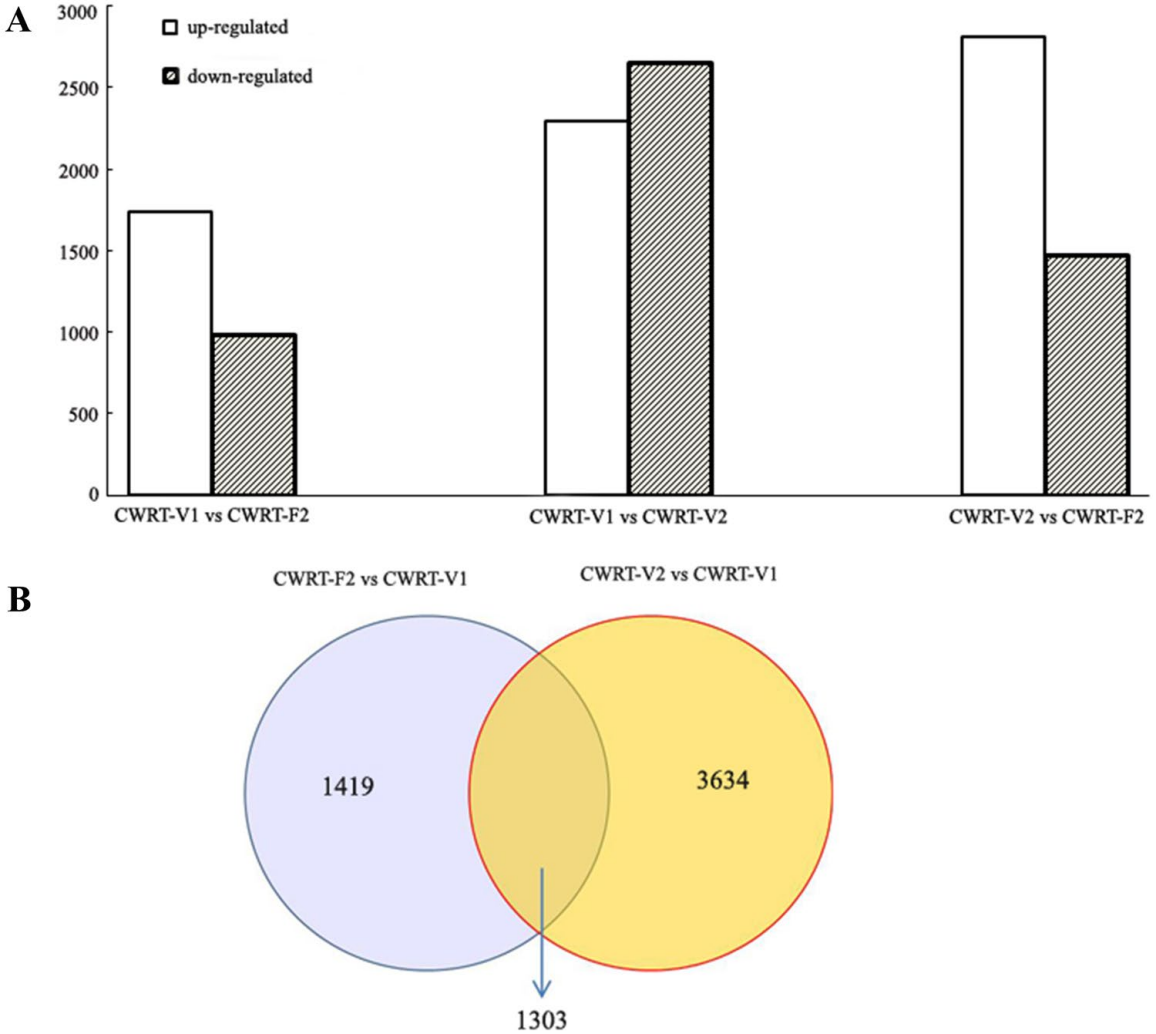
The statistics of differentially expressed genes. **a** Differentially expressed genes identified in the three libraries (CWRT-V1 vs. CWRT-F2; CWRT-V1 vs. CWRT-V2; CWRT-V2 vs. CWRT-F2); **b** Screen of differentially expressed genes related to flowering based on the original DGEs. The number in each cycle showed the differentially expressed gene number

After normalizing gene expression levels, we identified genes that had significant differences in expression between samples. First, we used the unigenes in CWRT-V1 as control. The DEGs were screened between the CWRT-V1 and CWRT-F2 libraries (Table S2). Between the CWRT-V1 and CWRT-F2 libraries, 1740 unigenes were significantly up-regulated, while 982 unigenes were significantly down-regulated. The up-regulated and down-regulated transcripts were shown in Fig. 1a. We also compared the gene expression levels between the CWRT-V1 and CWRT-V2 for each sub-group, and found that 2293 unigenes were significantly up-regulated, while 2644 unigenes were significantly down-regulated. Meanwhile, we used the unigenes in CWRT-V2 as control. In the double-flowering group, 2808 unigenes were significantly up-regulated, while 1461 unigenes were significantly down-regulated in CWRT-F2 library (Fig. 1a).

### Functional annotation of differentially expressed genes related to flowering

**Fig. 2:**
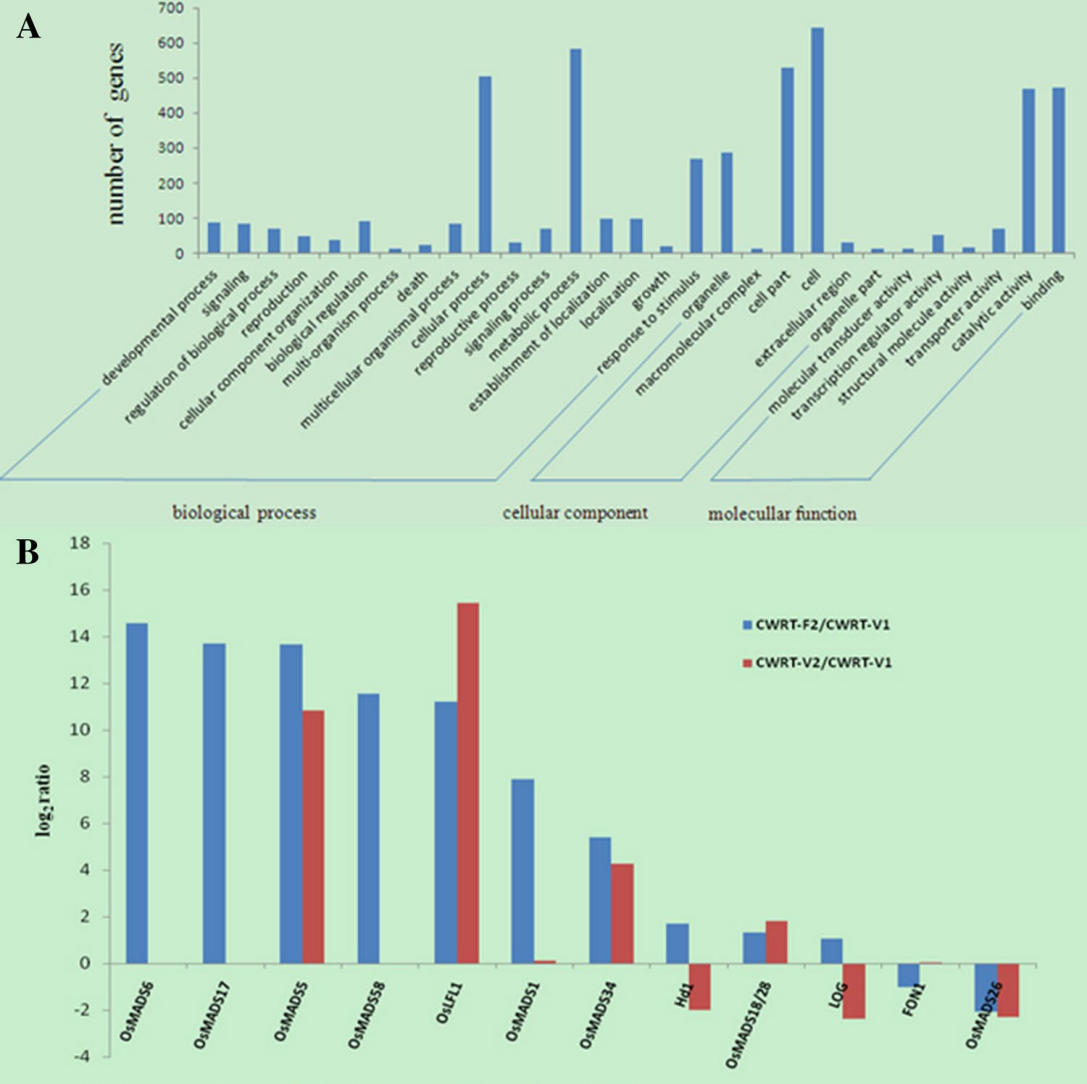
Expression analysis of genes involved in flowering development. **a** Histogram of gene ontology classification. The results are summarized in three main categories: biological processes, cellular components, and molecular function. The right y-axis indicates the number of genes in a category. The left y-axis indicates the percentage of a specific category of genes within the main category; **b** Expression pattern of 12 genes involved in flower transition and development

In order to identify flowering-related gene expression, we utilized the set of genes identified by DGE analysis for each of the three libraries. In theory, DGEs derived from comparison of CWRT-F2 vs CWRT-V1 represented only the genes that were expressed differentially between flowering and vegetative stages, as well as between different materials. Similarly, DGE gene sets derived from CWRT-V1 versus CWRT-V2 represented only the differentially expressed genes in the vegetative stages from the two sub-groups. Through comparison of DGE expression data between the V1 and V2 sub-groups, V2- and F2 sub-groups, and V1 and F2 sub-groups (Fig. 1b), we identified 1419 genes exclusively related to flowering in the double-flowering sub-group. KEGG pathway analysis and Go term annotation revealed that the 1419 unigenes fell into 119 KEGG signaling pathway classifications, and among them, 988 genes had GO term annotation (Fig. 2a). Go term analysis resulted in further classification of these genes into 29 sub-groups within three main categories: biological processes, cellular components, and molecular function. These groups accounted for approximately 45.8, 31.5 and 22.7% of the total unigenes, respectively.

### Identification of genes involved in flowering

MADS box genes are parts of a large family which regulates several types of developmental traits, including flower development in plants. To identify MADS box genes putatively involved in flower development, we screened the 1419 flowering-related genes. Through this heuristic search we found 12 genes involved in the flower transition and flower development (Fig. 2b). Among them, some were slightly up-regulated in CWRT-F2 compared with CWRT-V1, while they were slightly down-regulated in CWRT-V2, (including *OsMADS6, 17, 5, 58, 1*, 34), indicating these genes were related to flowering. Other genes, such as*OsMADS14* and *Hd1*, were strongly up-regulated in the CWRT-V1 library, indicating that these genes were related to floral meristem differentiation.

### Integrated analysis of miRNA and mRNA expression profiles

Forty-four differentially expressed miRNAs (17 up-regulated and 27 down-regulated) have been found to participate in flowering from CWRT-V1 and CWRT-F2 libraries (Chen et al. 2013). We performed a combined analysis using the 44 miRNAs, including 38 known and six new miRNAs, and the 1419 differentially expressed mRNAs identified in comparisons of the CWRT-V1 and CWRT-F2 libraries. As a result, 28 interacting miRNA-mRNA pairs showed antagonistic relationship, including miR156 and miR172 (Tables 2, S3). Of the miRNA-mRNA pairs, 23 known miRNAs belong to 12 families and five new miRNAs. In terms of correlated expression levels, 6 of the up-regulated miRNAs were negatively correlated with 27 of the down-regulated, target mRNAs, while 22 of the down-regulated miRNAs were negatively correlated with 66 up-regulated target mRNAs (Table 2).

**Table 2:** List of twenty-eight interacting miRNA–mRNA pairs

Number	miRNAs		Target genes	
	miRNA ID	Up/down^a^	Target gene-interaction	Up/down^a^
1	osa-miR156l	Up	*LOC_Os02g34860.1,LOC_Os03g26044.1, LOC_Os09g31970.1,LOC_Os04g34570.1, LOC_Os01g06550.1,LOC_Os07g29224.1, LOC_Os03g24960.1, LOC_Os06g05790.1*	Down
2	osa-miR530-5p	Up	*LOC_Os04g56980.1,LOC_Os03g17570.6, LOC_Os04g02880.1*	Down
3	oru-miR135	Up	*LOC_Os05g27930.1,LOC_Os01g67240.1, LOC_Os03g41080.1,LOC_Os07g38800.1, LOC_Os08g23180.1,LOC_Os11g45740.1*	Down
4	oru-miR139	Up	*LOC_Os01g10580.1,LOC_Os01g13760.1, LOC_Os01g32130.1*	Down
5	oru-miR177	Up	*LOC_Os09g39462.1*	Down
6	oru-miR180	Up	*LOC_Os06g24070.1,LOC_Os01g31890.1, LOC_Os01g31940.1,LOC_Os01g55450.1, LOC_Os02g47510.1, LOC_Os01g26000.1*	Down
7	osa-miR160f	Down	*LOC_Os04g43910.1,LOC_Os06g49840.1, LOC_Os02g39080.1, LOC_Os10g31864.1*	Up
8	osa-miR164a	Down	*LOC_Os05g18604.1,LOC_Os08g10080.1, LOC_Os01g62660.1,LOC_Os12g05260.1, LOC_Os09g08130.2,LOC_Os02g19924.1, LOC_Os06g46270.1,LOC_Os06g49660.1, LOC_Os09g37700.1,LOC_Os04g38720.1, LOC_Os12g41680.1*	Up
9	osa-miR164b	Down	*LOC_Os12g05260.1,LOC_Os06g46270.1, LOC_Os06g49660.1,LOC_Os04g38720.1*	Up
10	osa-miR164d	Down	*LOC_Os08g10080.1,LOC_Os12g05260.1, LOC_Os09g08130.2,LOC_Os06g46270.1, LOC_Os06g49660.1*	up
11	osa-miR164f	Down	*LOC_Os06g46270.1,LOC_Os12g41680.1, LOC_Os12g05260.1,LOC_Os04g41540.1*	Up
12	osa-miR166a	Down	*LOC_Os04g48290.1*	Up
13	osa-miR166b	Down	*LOC_Os08g34740.1*	Up
14	osa-miR166d	Down	*LOC_Os08g34740.1*	Up
15	osa-miR166f	Down	*LOC_Os08g34740.1*	Up
16	osa-miR166m	Down	*LOC_Os04g48290.1*	Up
17	osa-miR167d	Down	*LOC_Os06g03830.1*	Up
18	osa-miR167f	Down	*LOC_Os06g03830.1*	Up
19	osa-miR167h	Down	*LOC_Os03g29240.1*	Up
20	osa-miR172a	Down	*LOC_Os03g60430.2,LOC_Os03g47650.1 ,LOC_Os02g56320.1,LOC_Os06g49500.3, LOC_Os04g55560.4*	Up
21	osa-miR172b	Down	*LOC_Os04g55560.2*	Up
22	osa-miR172d	Down	*LOC_Os06g06050.1,LOC_Os04g05650.1*	Up
23	osa-miR2123c	Down	*LOC_Os02g49880.1*	Up
24	osa-miR390	Down	*LOC_Os02g10100.1,LOC_Os06g03970.1, LOC_Os01g33110.1,LOC_Os02g10100.1, LOC_Os05g33160.1,LOC_Os06g03970.1, LOC_Os04g45170.1*	Up
25	osa-miR408	Down	*LOC_Os03g15600.1,LOC_Os09g36860.1, LOC_Os08g37670.1,LOC_Os01g54430.1*	Up
26	osa-miR5161	Down	*LOC_Os05g47560.1,LOC_Os05g03884.1, LOC_Os11g01074.1,LOC_Os03g64320.1*	Up
27	osa-miR818b	Down	*LOC_Os01g10580.1,LOC_Os10g11200.1, LOC_Os02g54640.1,LOC_Os05g03640.1*	Up
28	oru-miR4	Down	*LOC_Os05g31530.1,LOC_Os09g39410.1*	Up

^a^CWRT-F2/CWRT-V1

In order to screen the miRNA-mRNA pairs for a role in regulation of flowering, target genes related to flowering were retrieved. In total, 12 miRNA-mRNA interacting pairs were identified that related to flowering (Table 3). Among them, five miRNAs (osa-miR156l, osa-miR160f, osa-miR172a, osa-miR172b, and osa-miR390), corresponding to nine target genes (*LOC_Os01g06550.1, LOC_Os02g34860.1, LOC_Os04g43910.1, LOC_Os06g49840.1, LOC_Os03g60430.2, LOC_Os04g55560.4, LOC_Os02g10100.1, LOC_Os06g03970.1*, and *LOC_Os01g33110.1*), have been reported to be involved in controlling flowering time. One gene (*LOC_Os01g62660.1*—a putative MYB family transcription factor), targeted by miR164, was up-regulated by miRNAs between CWRT-V1 and CWRT-F2 libraries.

**Table 3:** Integrative analysis of differentially expressed miRNAs that are negatively correlated with flowering-related genes from two databases (CWR-V1 and CWR-F2)

miRNA		Genes		
miRNA ID	F2/V1 up/down	Target gene-Interaction	F2/V1 up/down	Target gene description
osa-miR156l	Up	LOC_Os01g06550.1	Down	NF-X1-type zinc finger protein, putative
	Up	LOC_Os02g34860.1	Down	Regulator of chromosome condensation domain containing protein
osa-miR160f	Down	LOC_Os04g43910.1	Up	Auxin response factor, putative
	Down	LOC_Os06g49840.1	Up	OsMADS16 - MADS-box family gene with MIKCc type-box
osa-miR164a,b	Down	LOC_Os01g62660.1	Up	MYB family transcription factor, putative
osa-miR172a,b	Down	LOC_Os03g60430.2	Up	AP2 domain containing protein
	Down	LOC_Os04g55560.4	Up	AP2 domain containing protein
osa-miR390	Down	LOC_Os02g10100.1	Up	Leucine-rich repeat receptor protein kinase EXS precursor, putative
	Down	LOC_Os06g03970.1	Up	Receptor-like protein kinase 5 precursor, putative
	Down	LOC_Os01g33110.1	Up	Receptor-like protein kinase 5 precursor, putative
osa-miR408	Down	LOC_Os08g37670.1	Up	Plastocyanin-like domain containing protein, putative
	Down	LOC_Os01g54430.1	Up	Plastocyanin-like domain containing protein, putative
oru-miR135	Up	LOC_Os05g27930.1	Down	AP2 domain containing protein
	Up	LOC_Os01g67240.1	Down	Formin-like protein 1 precursor, putative
	Up	LOC_Os07g38800.1	Down	Lectin-like receptor kinase, putative
	Up	LOC_Os08g23180.1	Down	Fasciclin-like arabinogalactan protein 8 precursor, putative
	Up	LOC_Os11g45740.1	Down	MYB family transcription factor, putative
oru-miR139	Up	LOC_Os01g10580.1	Down	B-box zinc finger family protein, putative
oru-miR180	Up	LOC_Os06g24070.1	Down	myb-like DNA-binding domain containing protein
oru-miR4	Down	LOC_Os09g39410.1	Up	Male sterility protein, putative

*V1* CWRT-V1, *F2* CWRT-F2

In addition to analyze the interactions between known miRNAs and mRNAs, we also found 4 new miRNA-mRNA pairs involving in flowering. All of the four previously-unknown miRNAs were up-regulated during flowering stage, except ru-miR4 which was down-regulated. Two targets of oru-miR135 were down-regulated; these were LOC_Os05g27930.1, a predicted as AP2 domain containing protein, and LOC_Os11g45740.1, an MYB family transcription factor. A target (LOC_Os06g24070.1) of oru-miR180 was a predicted MYB-like DNA-binding domain containing protein. LOC_Os09g39410.1, a putative male sterility protein, was up-regulated by oru-miR4.

### Verification of the miRNA-mRNA interaction pairs

**Fig. 3:**
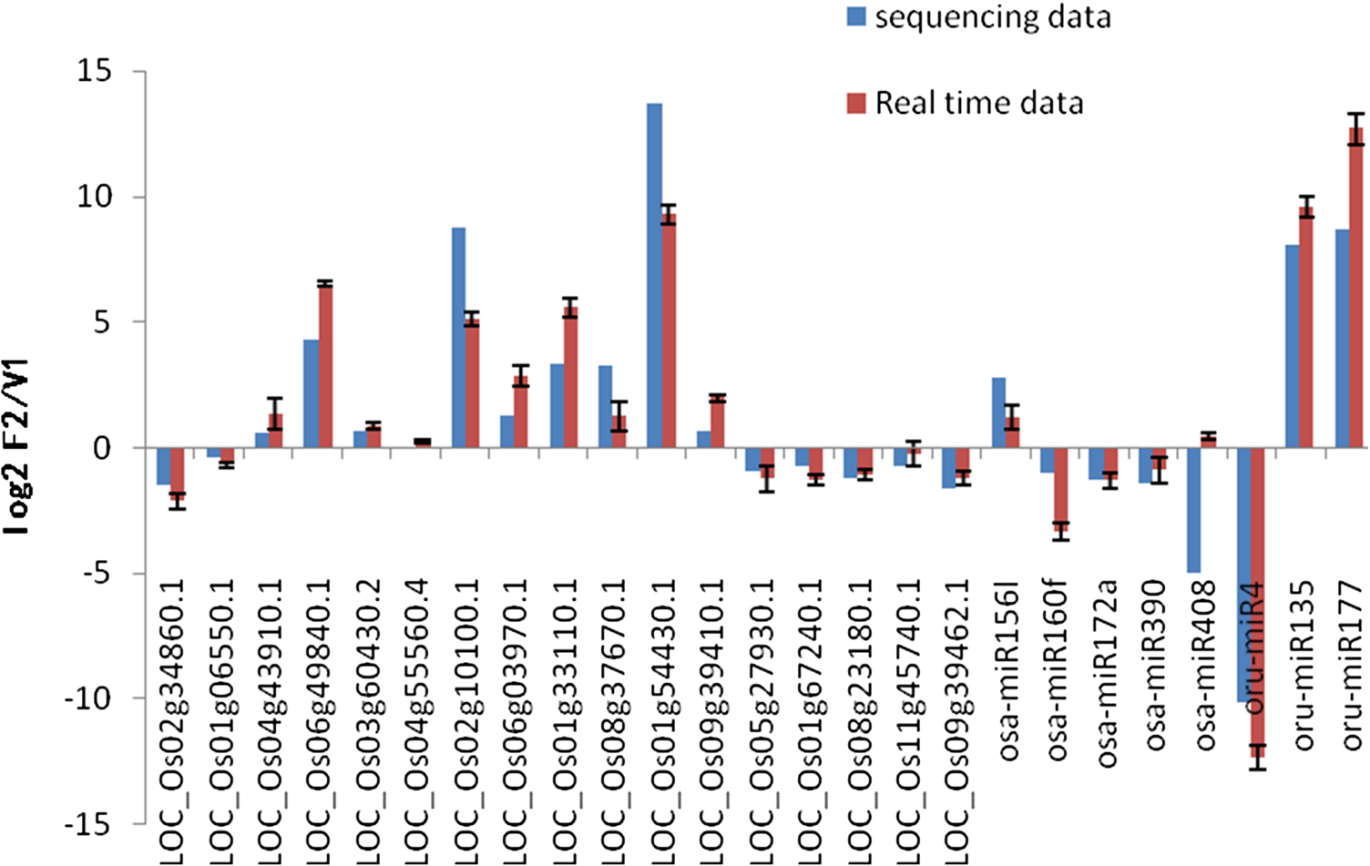
Validation of sequencing data by qRT-PCR. Twenty-five genes, including eight miRNAs and 17 mRNAs, were randomly selected and were subjected to quantitative real-time PCR analysis. The *U6* and *OsActin* were used as the internal references, respectively. Bars depict SD (n = 3). V*V1* CWRT-V1; *F2* CWRT-F2

Quantitative RT-PCR experiment was then performed to verify the relationship between miRNA and mRNA pairs. Eight differentially expressed miRNAs and 17 mRNAs were randomly selected for this qRT-PCR experiment. Comparison of the qPCR and sequencing data showed similar trends in gene expression for most miRNAs and mRNA, with the exception of some differences in fold changes (Fig. 3). We also calculated the relative expression levels between qPCR and sequencing data. The correlation values were highly significant with r = 0.90, indicating a high degree of accuracy for the high-throughput sequencing results.

## Discussion

Common wild rice is a useful resource for improvement of cultivated rice, with many potentially valuable genes for introgression. This work used next-generation sequencing technologies to investigate transcriptomic changes of a double-flowering common wild rice, compared with normal common wild rice, to discover flowering-related genes. 1419 differentially expressed mRNAs potentially involved in flowering were screened. Several flower meristem differentiation and flower stage developmental genes were up-regulated significantly in the reproductive stage. Besides DEGs, 44 differentially expressed miRNAs related to flowering time were identified in the previous study (Chen et al. 2013). After combining analysis, 28 miRNAs were found to regulate 93 mRNAs, indicating that miRNAs act in conjunction with other flowering time related genes to controlling flower development.

As one of the most extensively studied transcription factors in plants, the MADS-box family members were first identified in plants as floral homeotic genes. In previous studies, the comparison of expression profiles using a 22 k cDNA microarray-based transcriptome analysis of early panicle development in rice implicated three MADS-box genes, *OsMADS1, 14* and *15*, in panicle branching (Furutani et al. 2006). In this study, the above three genes were also identified and found to be involved in flowering. Besides these three genes, 16 other *OsMADS* genes were found to be potentially involved in flowering regulation and especially early flowering in common wild rice. Among them, some significantly up-regulated genes, including *OsMADS6, 17, 55, 58, 1*, 34 were related to flowering time, and *OsMADS14, 26, 18*/*28, 64* were related to floral transition. Besides the MADs box genes, other regulatory genes such as *Hd1* and *OsLFL1* have also been found related to tflowering.

In general, miRNAs negatively regulate their respective target genes. In this study, 28 miRNA-mRNA interaction pairs were matched, including miR156 and miR172. It has been reported that osa-miR156l, osa-miR160f, osa-miR172a, osa-miR172b, andosa-miR390 were involved in the control of flowering time (Spanudakis and Jackson 2014). We verified RNA-seq transcriptome data with qPCR of miRNA-mRNA interacting pairs. The correlation values were highly significant with r = 0.90, suggesting our approach of combined DGE and qPCR analyses with high-throughput sequencing yielded highly accurate data. In our analysis of miRNA-mRNA interaction pairs, the expression level of miR172 and miR156 were also different, which was consistent with previous reports (Poethig 2013). Furthermore, miR172 induces flowering by suppression of *OsIDS1* and *SNB*, two AP2 genes that negatively regulate expression of *Ehd1* and florigens, indicating that miRNAs and genes form a complex network to control flowering (Lee et al. 2014). Five new candidates were also identified besides the known miRNA-mRNA interaction pairs. Oru-miR135 appeared to target an AP2 domain-containing protein as well as an MYB family transcription factor. The AP2 domain-containing protein, like the miR172 targets*OsIDS1* and *SNB*, has been reported to suppress the transition from branch to spikelet meristem (Wang et al. 2015). Previous results have shown that the MYB family participating in anthocyanin biosynthesis (Vimolmangkang et al. 2013), flower development (Yan et al. 2014), and promotion of flowering (Abe et al. 2015; Zhang et al. 2016). Combined with previous studies and target descriptions for oru-miR135, we predict that oru-miR135 may target *LOC_Os05g27930.1* and *LOC_Os11g45740.1* to regulate flowering in CWRT-F2. Future studies will verify this hypothesis with experimental data. We also found that oru-miR180 targeted *LOC_Os06g24070.1*, a MYB-like DNA-binding domain containing gene, also related to flowering in CWRT-F2. In summary, the novel miRNA-mRNA interaction pairs were discovered to play a role in a complex regulatory network that controls flowering in common wild rice, and thus governs the double-flowering phenotype of the CWRT-F2 group.

The data provided here represent comprehensive and integrated genomic resources for cloning and identifying genes of interest in common wild rice. Characterization of the common wild rice transcriptome provides an effective tool for better understanding the molecular mechanisms of cellular processes and how miRNA regulates genes.

## Conclusions

In this study, we performed a large scale investigation to identify flowering-related genes in the common wild rice. DGE analysis revealed 1419 differentially expressed genes, including MADs box genes, potentially involved in flower transition and flower development. Subsequently, we integrated analysis of the miRNA and mRNA expression profiles. Twenty-eight miRNA-mRNA pairs were identified to control flowering. The data provided novel candidate genes and miRNAs related to flower development in common wild rice worthy of further investigations.

## Electronic supplementary material

Below is the link to the electronic supplementary material.

Supplementary material 1. Table S1 Primers used in this study. (XLSX 14 KB)

Supplementary material 2. Table S2 Differentially expressed genes identified in the three cDNA libraries. (XLSX 642 KB)

Supplementary material 3. Table S3 The miRNAs negatively correlated with their target genes from two databases (CWRT-V1 and CWRT-F2). (XLSX 17 KB)

## Abbreviations

CWRT-V1: Vegetative stage for single-flowering sub-group
CWRT-V2: Vegetative stage for double flowering sub-group
CWRT-F2: Flowering stage for double flowering sub-group
DGE: Digital gene expression
DEGs: differentially expressed genes
qRT-PCR: Quantitative reverse transcription PCR
